# Quality of life in older adults with chronic kidney disease and transient changes in renal function: Findings from the Oxford Renal cohort

**DOI:** 10.1371/journal.pone.0275572

**Published:** 2022-10-14

**Authors:** Isabella Busa, José M. Ordóñez-Mena, Yaling Yang, Jane Wolstenholme, Stavros Petrou, Clare J. Taylor, Chris A. O’Callaghan, Simon D. S. Fraser, Maarten W. Taal, Richard J. McManus, Jennifer A. Hirst, F. D. Richard Hobbs

**Affiliations:** 1 University of Oxford Medical School, Osler House, John Radcliffe Hospital, Oxford, United Kingdom; 2 Nuffield Department of Primary Care Health Science, University of Oxford, Oxford, United Kingdom; 3 National Institute for Health Research (NIHR) Oxford Biomedical Research Centre, Oxford University Hospitals NHS Foundation Trust, Oxford, United Kingdom; 4 Health Economics Research Centre, Nuffield Department of Population Health, University of Oxford, Oxford, United Kingdom; 5 Nuffield Department of Medicine, University of Oxford, Oxford, United Kingdom; 6 School of Primary Care, Population Sciences and Medical Education, Faculty of Medicine, University of Southampton, Southampton, United Kingdom; 7 Centre for Kidney Research and Innovation, School of Medicine, University of Nottingham, Nottingham, United Kingdom; The University of the West Indies, JAMAICA

## Abstract

**Background:**

Quality of life (QoL) is an important measure of disease burden and general health perception. The relationship between early chronic kidney disease (CKD) and QoL remains poorly understood. The Oxford Renal Study (OxRen) cohort comprises 1063 adults aged ≥60 years from UK primary care practices screened for early CKD, grouped according to existing or screen-detected CKD diagnoses, or biochemistry results indicative of reduced renal function (referred to as transient estimated glomerular filtration rate (eGFR) reduction).

**Objectives:**

This study aimed to compare QoL in participants known to have CKD at recruitment to those identified as having CKD through a screening programme.

**Methods:**

Health profile data and multi-attribute utility scores were reported for two generic questionnaires: 5-level EuroQol-5 Dimension (EQ-5D-5L) and ICEpop CAPability measure for Adults (ICECAP-A). QoL was compared between patients with existing and screen-detected CKD; those with transient eGFR reduction served as the reference group in univariable and multivariable linear regression.

**Results:**

Mean and standard deviation utility scores were not significantly different between the subgroups for EQ-5D-5L (screen-detected:0.785±0.156, n = 480, transient:0.779±0.157, n = 261, existing CKD:0.763±0.171, n = 322, p = 0.216) or ICECAP-A (screen-detected:0.909±0.094, transient:0.904±0.110, existing CKD:0.894±0.115, p = 0.200). Age, smoking status, and number of comorbidities were identified as independent predictors of QoL in this cohort.

**Conclusion:**

QoL of participants with existing CKD diagnoses was not significantly different from those with screen-detected CKD or transient eGFR reduction and was similar to UK mean scores for the same age, suggesting that patient burden of early CKD is minor. Moreover, CKD-related comorbidities contribute more significantly to disease burden in earlier stages of CKD than renal function *per se*. Larger prospective studies are required to define the relationship between QoL and CKD progression more precisely. These data also confirm the essentially asymptomatic nature of CKD, implying that routine screening or case finding are required to diagnose it.

## Introduction

Chronic Kidney Disease (CKD) is characterised by persistent decreased kidney function, defined by an estimated glomerular filtration rate (eGFR) of less than 60mL/min/1.73m^2^ [1], measured on at least 2 occasions at least 3 months apart, or by other evidence of kidney damage. CKD prevalence is increasing steadily with increasing prevalence of CKD risk factors such as obesity [2], diabetes [3], hypertension [4], and age [5]. In England, CKD prevalence is 18% for individuals aged over 60 years and costs the NHS an estimated £1.4 billion annually [6, 7]. Despite rising prevalence, CKD is often asymptomatic [8] and disease awareness remains alarmingly low [9]. Early CKD detection may facilitate timely intervention to prevent kidney function deterioration and reduce the overall burden of CKD-related kidney failure [10, 11].

Quality of life (QoL) measures the individual and societal burden of chronic diseases [12], and may serve as a surrogate measure of disease severity and progression. Health-related QoL (HRQoL) is a more focused assessment of general health perception [12–15]. Since both are measured in this study by the individual questionnaires, QoL is used as a generic term throughout.

Studies consistently indicate loss of function and well-being in people with CKD relative to the general population [12–14, 16–24], and reveal impaired QoL in people with more moderate renal insufficiency, some of whom lack formal diagnoses [14, 20, 25]. However, among people with CKD, no association has been reported between CKD severity and QoL; rather, QoL is poorer at all CKD stages compared to healthy individuals [13].

Nonetheless, it is likely that QoL may differ between participants with existing CKD diagnoses and those detected through screening. Since patients with comorbid conditions, including diabetes and hypertension, are routinely screened for CKD and renal impairment it is intuitive that these patients enriched for other comorbidities causative of organ damage would have a lower QoL than those detected at screening [26–28].

There are various measures of QoL. Studies using the Medical Outcomes Study Short Form-36 (SF-36) questionnaire report disproportionate impairment of physical QoL domains compared to mental QoL domains in adult people with CKD [12, 20]. The two outcome measures used in this study were collected as specific health economics outcome measures, used to value HRQoL and generic wellbeing and for use in economic evaluation when valued using their respective value weighted tariffs. By using multiple QoL scales, the suitability of either questionnaire for a CKD cohort can be evaluated and functional capability, well-being and general health perception of participants can be described in detail.

The 5-level EuroQol-5 Dimension (EQ-5D-5L) instrument measures HRQoL and comprises a health status classification system and a separate visual analogue scale (EQ-VAS) quantifying overall health perception [29, 30]. The ICEpop CAPability measure for Adults (ICECAP-A) [31] measures broader wellbeing than the EQ-5D-5L and other profile instruments focused on health status. The two questionnaires measure separate constructs and provide largely different, complementary information [32]. The ICECAP-A has not been widely used in the existing literature and, to our knowledge, never for a CKD cohort.

The primary aim of this investigation was to assess the relationship between QoL and early CKD in a screened cohort for the first time, by comparing people with existing CKD diagnoses with those identified through screening. Further interesting questions included whether any reduction in QoL is seen in people with transient changes to renal function and whether any observed changes in QoL were associated with reduced eGFR or comorbidity.

## Method

The Oxford Renal Study (OxRen) recruited individuals aged over 60 years from primary care practices in Oxfordshire, UK [7]. Baseline data from 1063 participants of the 3207 OxRen study invitees who had an established CKD diagnosis or a reduced eGFR measurement attended a baseline assessment until October 2020 were used. The sample size exceeds the population of 861 individuals recruited until July 2017 and included in the study by Hirst et al. [7] because continued follow-up and re-screening of the cohort meant that additional participants had one or more abnormal kidney function tests and thus became eligible for inclusion in this analysis.

Participants entered the cohort following existing CKD diagnoses in their general practice (GP) records (existing CKD) and screening tests of assessed serum creatinine to calculate eGFR using the older Chronic Kidney Disease Epidemiology Collaboration (CKD-EPI) formula, which corrects for ethnicity, and the urinary albumin:creatinine ratio (UACR). Positive results were as follows: CKD diagnosis in medical records, eGFR <60 ml/min/1.73m^2^, UACR >3mg/mmol; participants with 2 or more positive results were invited to baseline assessment.

Those with two positive OxRen screening tests at least 12 weeks apart and no existing CKD diagnosis were classified as having screen-detected CKD [33]. Those with only a single positive result in either of the two screening tests did not meet the full definition for CKD but were classified as having transient eGFR reduction, attended baseline assessment and represent the control group, since individuals who did not have an eGFR <60ml/min/1.73m^2^ at first screening were not followed up. The ‘transient eGFR reduction’ group therefore includes participants with transient (likely subclinical) AKI, but no background of chronic renal impairment in addition to any spurious abnormal results on first testing. The study protocol was approved by South Central Oxfordshire Research Ethics Committee B and registered with the UK Clinical Research Network. All study participants gave written informed consent prior to recruitment.

At the baseline visits, participants were comprehensively characterised: QoL, clinical and demographic data were obtained at baseline assessment using two QoL questionnaires, clinical testing, and detailed medical histories. The case report form used at the baseline assessment to collect these data and define relevant comorbid conditions is included (**S1 Appendix**). ‘Number of comorbidities’ was calculated as a cumulative score of comorbid conditions identified at baseline visit for each participant.

Each of the five dimensions of health status in the EQ-5D-5L questionnaire were scored on five levels (from 1 to 5): no, slight, moderate, severe, and extreme problems. Participants also gave one number indicative of their overall health on the separate EQ-VAS scale anchored at 0 (worst imaginable health) and 100 (best imaginable health). Participants were required to both indicate their score on the scale and write their response in a box. Where there was a discrepancy between these scores, the number in the box was given priority.

Responses to the EQ-5D-5L descriptive system were valued using the cross-walk mapping algorithm onto the EQ-5D-3L value set in accordance with NICE’s position statement [34], with values indexed at 0 (equivalent to death) and 1 (perfect health). EQ-5D-5L utility scores were analysed as the primary outcome measure since they are a comprehensive measure of overall QoL, incorporating scores for all EQ-5D-5L domains and EQ-VAS.

For each of the five items of capability in the ICECAP-A scoring comprises four response levels (from 1 to 4): no, little, much, and full capability. ICECAP-A scores for all attributes were also combined into utility scores anchored at 0 (no capability) and 1 (full capability) using a UK general population tariff [35].

### Statistical analysis

All data management and analyses were performed using R (version 4.0.3) [36]. Data were tabulated for the full cohort and stratified by the three prespecified CKD subgroups (existing CKD, screen-detected CKD, transient GFR reduction). Categorical variables, summarised as frequencies and percentages, were compared between subgroups using Pearson’s chi-squared test. Means and standard deviations were used to summarise all continuous variables, and subgroups compared using ANOVA. Where these data were non-normally distributed, logarithmic transformation of the data and Kruskal-Wallis non-parametric testing was used.

Multiple linear regression was used to estimate beta coefficients (difference in mean QoL utility scores) and 95% confidence intervals (CI) for the association of CKD subgroups and pre-specified demographic clinical variables with QoL utility score. Only those comorbidities shown to be relevant to QoL of CKD patients in existing literature [18, 32, 37] were adjusted for initially; Model 1 adjusted for age, sex, BMI, diabetes, hypertension, CVD, smoking status, eGFR, and number of comorbidities as a primary set of confounding variables (Model 1).

Model 2 adjusted for all demographic and clinical data collected in OxRen (primary variables plus ethnicity, obesity, hip circumference, systolic and diastolic blood pressure, alcohol consumption, history of heart failure, atrial fibrillation, cerebral and peripheral vascular diseases, previous renal disease such as renal stones or cysts (which are separate from, but may contribute to, CKD), urinary tract infection, thyroid problems, anaemia, osteoporosis, osteopenia, and education status). Number of comorbidities was excluded from Model 2 due to multicollinearity with each of the comorbid conditions included as individual covariates. Obesity, anaemia, and hypertension were dichotomised such that participants either ‘had’ or ‘did not have’ the condition according to clinically relevant thresholds. QoL scores were also adjusted for CKD stage in an independent regression model. CKD stage was defined according to the 2012 KDIGO guidelines [38].

Missing QoL values were reported but excluded from further analysis; there were no missing data for demographic covariates. Multicollinearity was assessed with the variance inflation factor. The significance threshold was defined as 0.05 throughout.

## Results

### Descriptive analyses

Of the 1063 participants assessed at baseline assessment, 322 participants had existing CKD diagnoses (30.3%), 480 participants were diagnosed through screening (45.2%) and 261 participants had transient eGFR reduction (24.5%). 926 completed the EQ-5D-5L questionnaire, including EQ-VAS (87.1% completion) and 888 individuals answered all dimensions of the ICECAP-A questionnaire (83.5% completion rate). 857 participants had data for both questionnaires (**S1 Fig**).

**Table 1** characterises the 857 participants at baseline assessment with complete QoL data, stratified by CKD subgroup. **S1 Table in S1 File** summarises the entire OxRen cohort, including those participants with missing or incomplete QoL data.

**Table 1:** Baseline characteristics of the OxRen cohort of older adults from Oxfordshire primary care practices with complete QoL data.

	Entire cohort (n = 857)	Existing CKD (n = 272)	Screen-detected CKD (n = 358)	Transient eGFR reduction (n = 227)	p-value^a^
Characteristics	Mean (SD)	Mean (SD)	Mean (SD)	Mean (SD)	
Age (years)	74.52 (6.78)	75.68 (6.94)	74.28 (6.37)	73.51 (7.02)	0.001*
Weight (kg)	77.81 (16.74)	77.49 (16.56)	77.83 (16.93)	78.15 (16.71)	0.906
Height (m)	1.67 (0.10)	1.67 (0.10)	1.68 (0.10)	1.67 (0.10)	0.283
Waist circ. (cm)	96.80 (14.53)	97.21 (13.67)	96.26 (15.20)	97.14 (14.50)	0.662
Hip circ. (cm)	106.23 (10.43)	107.05 (10.25)	105.37 (10.60)	106.60 (10.31)	0.113
BMI (kg/m^2^)	27.70 (5.27)	27.77 (5.02)	27.54 (5.60)	27.86 (5.04)	0.750
eGFR (ml/min/1.73m^2^)	63.79 (15.46)	56.15 (14.80)	67.64 (14.38)	66.89 (14.57)	<0.001*
Number of comorbidities	2.37 (1.46)	3.07 (1.37)	2.00 (1.41)	2.13 (1.37)	<0.001*
**Sex**	**n (%)**	**n (%)**	**n (%)**	**n (%)**	
Male	400 (46.67)	119 (43.75)	184 (51.40)	97 (42.73)	0.067
Female	456 (53.21)	153 (56.25)	174 (48.60)	129 (56.83)	
**Ethnicity**					
White	98.60%	98.90%	98.60%	98.24%	0.947
Other	1.28%	1.10%	1.40%	1.32%	
**Comorbid disease**					
CKD stage II	506 (61.19)	108 (46.75)	261 (69.60)	137 (61.99)	<0.001*
CKD stage IIIa	277 (33.49)	111 (48.05)	94 (25.07)	72 (32.58)	<0.001*
CKD stage IIIb—IV	44 (5.32)	12 (5.19)	20 (5.33)	12 (5.43)	0.497
Hypertension	503 (58.69)	180 (66.18)	193 (53.91)	130 (57.27)	0.007*
Diabetes	127 (14.82)	61 (22.43)	35 (9.78)	31 (13.66)	<0.001*
Ischaemic Heart Disease	154 (17.97)	55 (20.22)	56 (15.64)	43 (18.94)	0.302
Heart failure	37 (4.32)	15 (5.51)	15 (4.19)	7 (3.08)	0.408
Atrial Fibrillation	107 (12.49)	41 (15.07)	39 (10.89)	27 (11.89)	0.277
Cerebrovascular disease	72 (8.40)	26 (9.56)	32 (8.94)	14 (6.17)	0.354
Peripheral vascular disease	31 (3.62)	8 (2.94)	14 (3.91)	9 (3.96)	0.770
Previous Renal Disease	341 (39.79)	236 (86.76)	71 (19.83)	34 (14.98)	<0.001*
Anaemia	99 (11.55)	34 (12.50)	38 (10.61)	27 (11.89)	0.751
Osteoporosis	72 (8.40)	24 (8.82)	32 (8.94)	16 (7.05)	0.692
Osteopenia	47 (5.48)	17 (6.25)	22 (6.15)	8 (3.52)	0.318
**Smoking status**					
Never	461 (53.79)	139 (51.10)	191 (53.35)	131 (57.71)	0.429
Former	357 (41.66)	121 (44.49)	147 (41.06)	89 (39.21)	
Current	39 (4.55)	12 (4.41)	20 (5.59)	7 (3.08)	
**Educational status**					
No qualifications	322 (37.57)	105 (38.60)	138 (38.55)	79 (34.80)	0.656
GCSE/O-levels	148 (17.27)	45 (16.54)	65 (18.16)	38 (16.74)	
A-levels	168 (19.60)	48 (17.65)	66 (18.44)	54 (23.79)	
University/ Postgraduate	219 (25.55)	74 (27.21)	89 (24.86)	56 (24.67)	

^a^ Variables compared between CKD subgroups

*p<0.05.

The subset of participants with complete QoL (**Table 1**) had a female majority (53.2%) and was predominantly white (98.6%). Hypertension (58.7%) and previous renal disease (39.8%) were the most common previously known comorbidities in the cohort. Participants with existing CKD diagnoses were older (75.7±6.9 years) than those with screen-detected (74.3±6.4 years) and transient GFR reduction (73.5±7.0years) (p = 0.001). Hypertension (p<0.01), diabetes (p<0.001), and previous renal disease (p<0.001) were more common in participants with existing CKD diagnoses compared with the other subgroups. While these subgroup differences are all statistically significant, further post-hoc analysis is required to visualise the specific differences between subgroups.

As expected, participants with existing CKD diagnoses had more comorbid conditions (3.07±1.37) than other participants (screen-detected: 2.00±1.37, transient GFR reduction: 2.13±1.37, p<0.001). eGFR stratified similarly: individuals with existing CKD had significantly worse eGFR at baseline (56.15±14.80ml/min/1.73m^2^) than individuals in the other CKD subgroups (screen-detected: 67.64±14.38ml/min/1.73m^2^, transient GFR reduction: 66.89±14.57ml/min/1.73m^2^, p<0.001). These differences in eGFR likely underlie the significant differences in CKD stages II and IIIa between subgroups.

**S2 Table in S1 File** compares participants with incomplete and complete QoL data. Only previous renal disease and number of comorbidities differed between the subsets; this, in combination with further analysis of only those participants with complete QoL data, reassures against systematic differences and justifies the generalisation of subsequent analyses to include those with incomplete QoL data.

### Quality of life domains

**Fig 1:**
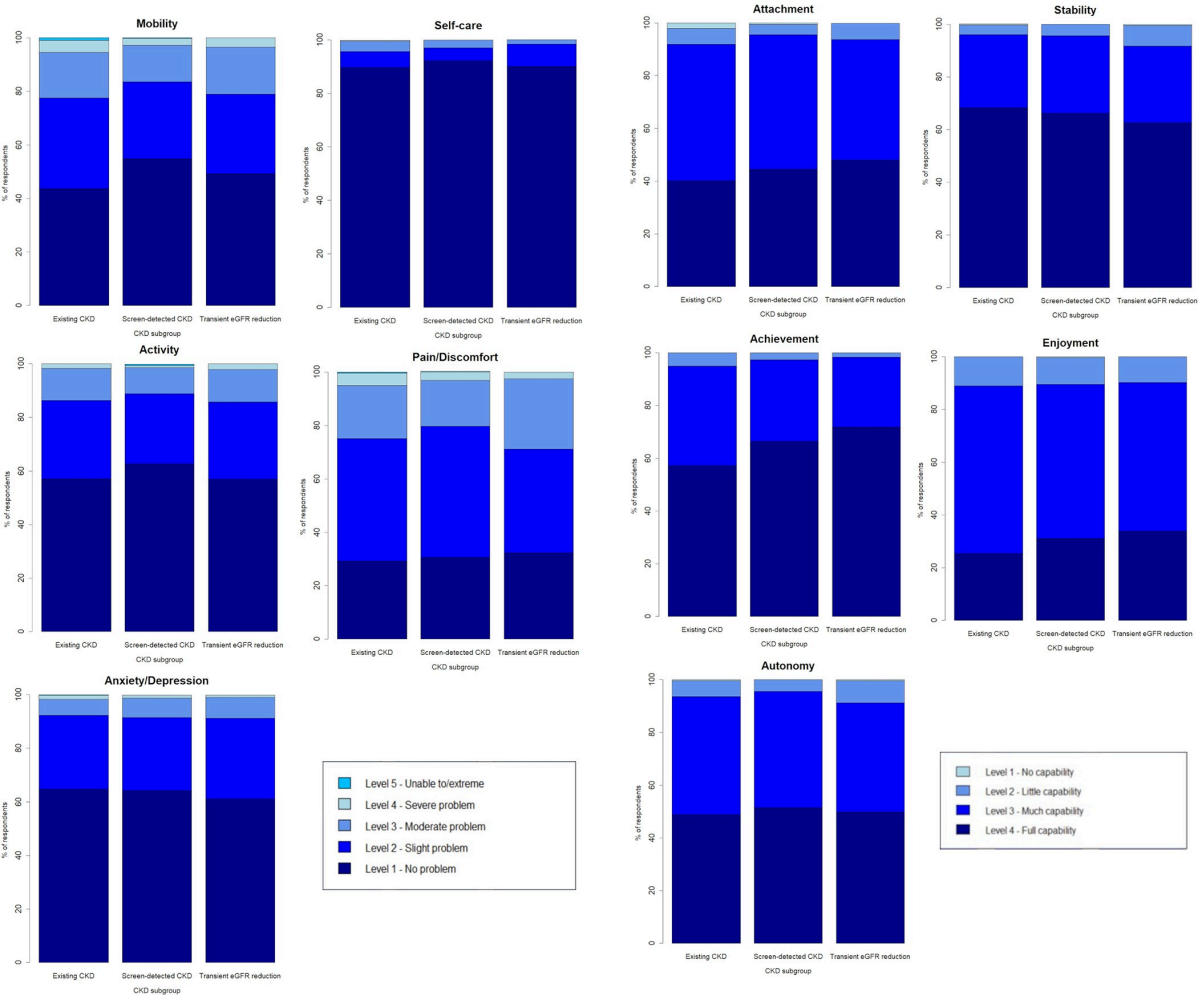
EQ-5D-5L (a) and ICECAP-A (b) questionnaire responses stratified by dimension.

**Fig 1** presents EQ-5D-5L (**Fig 1A**) and ICECAP-A (**Fig 1B**) QoL data stratified by questionnaire dimension and by CKD subgroup. These data are tabulated in **S3 Table in S1 File**. EQ-5D-5L highlighted self-care as the least affected (‘no problem’ for 90.8% of entire cohort), and pain/discomfort the most affected QoL domain (‘no problem’ for 30.5% of entire cohort). Participants with transient GFR reduction reported the lowest impact on pain/discomfort (‘no problem’ for 32.2% compared to 30.5% and 29.2% for those with screen-detected and existing CKD respectively). Screen-detected participants had the highest proportion reporting no problems for mobility (54.8%) and self-care (92.2%) compared with transient eGFR reduction (mobility 49.2% and self-care 90.0%) and existing CKD (mobility 43.6% and self-care 89.6%).

ICECAP-A highlighted Enjoyment and Attachment as the two attributes most impacted in this CKD cohort (**Fig 1B, S3 Table in S1 File**). Only 30.0% of participants reported full Enjoyment capability (level 4), with this domain was consistently impaired across subgroups. Individuals with transient eGFR reduction were the least impaired in the Enjoyment and Attachment domains (47.9% of this subgroup reported full Attachment capability compared to 44% for the entire cohort). Achievement was shown to be the least affected dimension (96.9% full or much capability), and was most affected in those with existing CKD, while remaining dimensions (Stability 65.9% and Autonomy 50.2% full capability) were not notably impacted by CKD in any subgroup.

**Table 2** shows mean questionnaire scores for the three QoL questionnaires, stratified by CKD subgroup. Mean (±SD) EQ-VAS score for the CKD cohort was 79.2±15.3; there was no statistically significant difference between the subgroups in ANOVA testing (p = 0.78), non-parametric testing (p = 0.83), and logarithmic transformation of questionnaire scores (p = 0.91). There were also no significant differences between CKD subgroups in QoL scores combining all dimensions for either EQ-5D-5L or ICECAP-A questionnaires.

**Table 2:** Mean questionnaire scores in the entire OxRen cohort of older adults from Oxfordshire primary care practices and stratified by CKD subgroups.

	Entire cohort (n = 1063)	Existing CKD (n = 322)	Screen-detected CKD (n = 480)	Transient eGFR reduction (n = 261)	p-value
EQ-VAS Mean (SD)	79.2 (15.3)	79.0 (15.7)	79.6 (15.2)	78.7 (14.9)	0.781
EQ-5D-5L Utility Mean (SD)	0.776 (0.161)	0.763 (0.171)	0.785 (0.156)	0.779 (0.157)	0.216
ICECAP-A Utility Mean (SD)	0.903 (0.105)	0.894 (0.115)	0.909 (0.094)	0.904 (0.110)	0.200

### Association of CKD subgroups and other predictors with quality of life utility scores

**Table 3** shows differences in mean EQ-5D-5L and ICECAP-A utility scores by CKD subgroup (existing, screen-detected, or transient) and adjustment variables. There were no significant differences in mean QoL between CKD subgroups, regardless of the level of adjustment of type of QoL questionnaire.

**Table 3:** Unadjusted and adjusted estimates of the association of CKD subgroup and other covariates with QoL utility score.

	N	Mean Utility Score	Unadjusted^a^ analysis	Adjusted analyses	
				Model 1^b^	Model 2^c^
			Coefficient (CI)	Coefficient (CI)	Coefficient (CI)
**EQ-5D-5L**					
**Existing CKD diagnosis**	480	0.763	0 Reference	0 Reference	0 Reference
**Screen-detected CKD**	322	0.785	0.022 (-0.003, 0.046)	0.007 (-0.017, 0.031)	0.003 (-0.028, 0.034)
**Transient eGFR reduction**	261	0.779	0.016 (-0.011, 0.044)	0.001 (-0.026, 0.027)	-0.001 (-0.035, 0.032)
**Age (per 1 year)**	1063	0.776	-0.004* (-0.005, -0.002)	-0.004* (-0.006, -0.003)	-0.004* (-0.006, -0.002)
**Female vs male sex**	488	0.785	-0.017 (-0.038, 0.004)	-0.036* (-0.057, -0.015)	-0.046* (-0.084, -0.009)
**BMI (per 1 kg/m** ^**2**^ **)**	1063	0.776	-0.005* (-0.007, -0.003)	-0.005* (-0.007, -0.003)	-0.009 (-0.021, 0.004)
**Diabetes**	163	0.728	-0.057* (-0.086, -0.028)	-0.038* (-0.067, -0.008)	-0.032* (-0.063, -0.001)
**Hypertension**	615	0.768	-0.019 (-0.041, 0.002)	0.001 (-0.020, 0.023)	0.002 (-0.021, 0.024)
**Ischaemic heart disease**	186	0.747	-0.035* (-0.063, -0.008)	-0.021 (-0.048, 0.006)	-0.005 (-0.034, 0.024)
**Never smoked**	579	0.796	0 Reference	0 Reference	0 Reference
**Former smoker**	438	0.755	-0.041* (-0.063, -0.020)	-0.038* (-0.059, -0.017)	-0.023* (-0.046, -0.000)
**Current smoker**	46	0.721	-0.076* (-0.013, -0.024)	-0.099* (-0.150, -.0485)	-0.097* (-0.150, -0.043)
**eGFR (per 1 ml/min/1.73m** ^**2**^ **)**	1063	0.776	0.001* (0.001, 0.002)	0.001 (0.000, 0.001)	0.000 (-0.001, 0.001)
**Number of comorbidities**	1063	0.776	-0.028* (-0.034, -0.021)	-0.029* (-0.039, -0.018)	
**ICECAP-A**					
**Existing CKD diagnosis**	480	0.895	0 Reference	0 Reference	0 Reference
**Screen- detected CKD**	322	0.909	0.015 (-0.001, 0.031)	0.010 (-0.007, 0.026)	0.008 (-0.015, 0.030)
**Transient eGFR reduction**	261	0.904	0.010 (-0.008, 0.028)	0.005 (-0.013, 0.024)	0.002 (-0.022, 0.026)
**Age (per 1 year)**	1063	0.903	-0.001* (-0.002, 0.000)	-0.001* (-0.001, -0.002)	-0.002 (-0.002, -0.001)
**Female vs male sex**	488	0.911	-0.014* (-0.028, 0.000)	-0.022* (-0.036, 0.007)	-0.021 (-0.047, 0.004)
**BMI (per 1 kg/m** ^**2**^ **)**	1063	0.903	-0.001* (-0.003, 0.000)	-0.001* (-0.001, -0.003)	-0.005 (-0.010, 0.001)
**Diabetes**	163	0.895	-0.010* (-0.030, 0.009)	-0.003 (-0.023, 0.018)	-0.007 (-0.029 to 0.015)
**Hypertension**	615	0.900	-0.008 (-0.023, 0.006)	-0.002 (-0.017, 0.012)	0.000 (-0.016, 0.024)
**Ischaemic heart disease**	186	0.885	-0.023* (-0.041, -0.005)	-0.021* (-0.039, 0.002)	-0.005 (-0.034, 0.016)
**Never smoked**	576	0.911	0 Reference	0 Reference	0 Reference
**Former smoker**	438	0.896	-0.015* (-0.030, -0.001)	-0.016* (-0.030, -0.001)	-0.012 (-0.028, 0.004)
**Current smoker**	46	0.875	-0.036* (-0.071, -0.002)	-0.045* (-0.080, -0.010)	-0.042* (-0.079, -0.005)
**eGFR (per 1 ml/min/1.73m** ^**2**^ **)**	1063	0.903	0.001* (0.000, 0.001)	0.000 (0.000, 0.001)	0.000 (0.000, 0.001)
**Number of comorbidities**	1063	0.903	-0.010* (-0.015, -0.005)	-0.008 (-0.015, 0.000)	

^a^Unadjusted: simple linear regression

^b^Model 1: multiple linear regression, partially adjusted for primary covariates

^c^Model 2: multiple linear regression, fully adjusted for all clinical and demographic variables.

*p<0.05.

Older age, female sex, former and current smoking status were associated with lower mean EQ-5D-5L utility score in unadjusted and adjusted analyses. Increased eGFR and BMI, and a history of ischaemic heart disease were associated with lower mean EQ-5D-5L score in unadjusted but not adjusted analyses. The results for ICECAP-A were consistent for current smoking status only (-0.044, CI:-0.079 to -0.009). All other covariates were not associated with mean ICECAP-A score across models with different degrees of adjustment.

Increased number of comorbidities was associated with decreased mean EQ-5D-5L utility score in both unadjusted (-0.028, CI: -0.034 to -0.021) and partially adjusted (-0.029, CI: -0.039 to -0.018) analysis. Number of comorbidities was similarly associated with ICECAP-A utility score in unadjusted (-0.010, CI: -0.015 to -0.005), but not adjusted analysis.

Adjustment for CKD stage in a separate regression model (**S5 Table in S1 File**) showed CKD stages IIIa and IIIb were independently associated with EQ-5D-5L utility score. There were no other statistically significant associations between CKD stage and QoL stage.

Sensitivity analyses (**S6 Table in S1 File**) revealed strong collinearity for height, weight, and waist circumference, marking these factors as redundant in the presence of other variables; they were removed from the regression model with BMI maintained as an index of these variables. All regression assumptions were satisfied for all analyses, barring normality of the residuals. Logarithmic and logistic transformation improved normality and corroborated the statistical significance of untransformed QoL utility scores.

## Discussion

Overall, this population-based cohort study of English primary care patients screened for CKD found no statistically significant difference in QoL, as measured by two different questionnaires, between participants with existing CKD diagnoses, screen-detected CKD, and a transient eGFR reduction. The finding that number of comorbidities was associated with QoL in adjusted regression analyses of EQ-5D-5L scores is the most important finding in this study. Other factors independently associated with impaired QoL included increased age and current smoking status. Neither eGFR nor BMI, which was in the overweight range for all subgroups, were associated with QoL impairment, regardless of the questionnaire used. This is the first study, to our knowledge, to examine QoL in a primary care population taking part in a CKD screening programme and the first to use ICECAP-A for a CKD cohort.

It is interesting that neither eGFR nor CKD subgroup were independently associated with QoL in this study, and that there was only limited association between CKD stage and QoL score. It is possible that the statistical power of this study was insufficient to detect any difference between the CKD subgroups since the cohort was generally healthy and disease progression is typically asymptomatic. It is also notable that mean eGFR for the screen-detected CKD subgroup is greater than the 60ml/min/1.73m^2^ diagnostic cut-off; it suggests that CKD as defined by UACR constitutes a substantial proportion of CKD diagnoses made in OxRen. Overall, these findings suggest that subtle differences in CKD status between participants are not manifested in differential health perception.

Mean EQ-5D-5L utility score (0.776±0.161) for this cohort aged over 60 years corroborates population averages [15], reported as 0.856 for the UK general population, reduced to 0.785 for individuals aged 65–74 years and 0.734 over 75 years [15]. The EQ-VAS UK population norm is given as 80.0, reduced to 77.3 and 73.8 for 65–74 and ≥75 years respectively [15]. Mean EQ-VAS score in this study (79.2±15.3) indicates no significant impairment in people with early stages of CKD compared to the general population at the same age.

The distribution of both EQ-5D-5L and ICECAP-A scores showed a ceiling effect, whereby a high proportion of participants have a maximum utility score (1.00). This combined with the high mean utility score for ICECAP-A (0.903±0.105) suggest this questionnaire is inadequately sensitive to subtle QoL impairments. Since early CKD is typically asymptomatic, it is intuitive that capability would not be impaired in this cohort.

In this study, number of comorbidities was associated with QoL impairment in adjusted analyses of EQ-5D-5L but not ICECAP-A scores. The two questionnaires are not directly comparable since they have different theoretical bases and measure qualitatively different aspects of QoL. Several studies in elderly populations support the complementary use of these questionnaires in combination [32, 39–41]. Since ICECAP-A captures more QoL dimensions than traditional HRQoL instruments in Type 2 Diabetes patients [42] and is more suitable than EQ-5D-5L for assessing adults with psychiatric conditions [43], it may be that ICECAP-A becomes more relevant for this CKD cohort with disease progression.

### Strengths and limitations

This study assessed multiple QoL scales, permitting evaluation of questionnaire suitability for a CKD cohort and detailed description of functional capability, well-being, and general health perception. It is the first study, to our knowledge, to use ICECAP-A for this disease cohort and to assess QoL in a screening programme of early, pre-dialysis CKD. Reporting both dimension-by-dimension and utility scores, the study maximises opportunity for future analysis of the OxRen cohort and overcomes the limitations of either questionnaire used in isolation. Extensive baseline demographic and clinical data collected in this study facilitated adjustment for a range of potentially confounding variables.

Reporting data at baseline prevented precise investigation of the relationship between CKD progression and QoL impairment in the present study. There is also potential for residual confounding by variables neither measured nor adjusted for in this study; number and type of medications is a relevant missed covariate since it is likely to affect both physical and mental aspects of QoL and be differentially distributed between CKD subgroups [44]. The significant impact dialysis is known to have on QoL [45] has also not been considered, nor has marital status. Additionally, since many of the covariates tested in this study were binary (participants either ‘had’ or ‘did not have’ comorbid conditions such as anaemia, obesity, and hypertension) any difference in severity between individuals and CKD subgroups is not accounted for.

Selection bias is also possible since OxRen recruitment relied on volunteers following an invitation. OxRen did not collect QoL data for screen-positive patients prior to being told their diagnosis and it is likely that individuals with better QoL are more able, and more likely, to participate in a study; this may have confounded the lack of correlation between neither eGFR nor CKD with QoL in this cohort. The impact of chronic disease diagnoses on patients is important: one hypertension screening programme similar to OxRen saw a significant increase in absenteeism in the year immediately after screening as a result of participants knowing their diagnosis [46].

Non-white ethnic groups were under-represented in our study (2% vs 5% in the 2011 census for individuals over 60 years [47]). EQ-VAS, but not EQ-5D-3L, scores were found to be statistically significantly lower among south Asians with diabetes compared to a white European subgroup in one study [48]. Although no significant relationship between ethnicity and EQ-5D-5L utility score was identified in the present investigation, it is possible that the ethnicity of the cohort may have biased the results, improving QoL scores to higher than is representative of the general population. However, this over-representation of Caucasians in the study population was not deemed significant enough to undermine comparison with UK population averages.

### Comparison with existing literature

QoL impairments in CKD patients relative to healthy individuals are consistently reported [12–14, 16–23] However, no association has been observed between CKD severity and overall QoL in the present study; this is at odds with existing studies showing an association between eGFR and QoL [16, 18, 20]. Although the finding that physical EQ-5D-5L dimensions were disproportionately impaired corroborates existing conclusions [14, 22, 24, 39], this may be explained by the finding that physical QoL deteriorates over time while mental QoL remains stable over a longer period [45].

The SF-36 questionnaire is most commonly used in studies of CKD [13, 14, 16, 49, 50], especially of paediatric [23, 51–54] and kidney transplant populations [23, 26, 44]. Studies investigating QoL in pre-dialysis CKD individuals [13, 22] have focused on the relationship between QoL and CKD progression, identifying low QoL as an independent risk factor of CKD progression [22]. Overall, the disparities in patient cohort, QoL questionnaire, stage of disease, and research focus between this study and the existing literature have not contributed to a significant difference in findings, and this study largely supports existing conclusions.

Age, sex, history of cardiovascular event, diabetes, anaemia, obesity, and hypertension are consistently reported as independent risk factors of QoL in both adult [13, 37, 55] and elderly [16] CKD patients. Predictors of QoL identified in this study included age and smoking status; neither hypertension, obesity nor anaemia were independently associated with QoL. This contrasts findings that decreased haemoglobin concentrations [12, 13, 16] and obesity [55–57] independently predict low QoL in CKD, although it remains unclear whether the anaemia and obesity here are equivalent to existing literature. Nevertheless, the total number of comorbidities was independently associated with lower QoL, suggesting that it is medical conditions associated with CKD rather than low eGFR per se that contribute to lower QoL in persons with early stage CKD. This is important because CKD typically occurs in the context of multimorbidity.

The observation that pain/discomfort and self-care were the EQ-5D-5L dimensions most and least affected by CKD in this cohort respectively corroborates existing observations. In a 2013 study, 48.3% of the UK population reported ‘problems’ in pain/discomfort, compared to just 3.7% for self-care [15]. It is possible that the present results may reflect qualities inherent to EQ-5D questionnaire design rather than specific characteristics of the CKD cohort. Future studies utilising other QoL scales are required to verify the extent to which results may have been influenced by the questionnaires themselves.

### Clinical implications

Overall, QoL impairments in this cohort are slight relative to population norms, indicating that patient burden of early CKD is minor. Nevertheless, it remains possible that QoL declines with eGFR in individuals, therefore prospective studies are required to investigate changes in QoL with CKD progression within individuals; continued data collection in OxRen represents a good opportunity to do this.

Ultimately, it is likely that CKD-related comorbidities contribute more significantly to disease burden in earlier stages of CKD than renal function per se. The finding that number of comorbidities was associated with QoL in adjusted regression analyses of EQ-5D-5L scores, and that eGFR had very little effect, is the most important finding in this study. However, that there was no difference in QoL between the CKD subgroups in a fully adjusted model, despite eGFR and number of comorbid conditions differing between the subgroups, suggests that any observed QoL impairment in these individuals with pre-dialysis CKD is more complex than may be attributable to any one variable. It is also notable that current smoking status was identified as an important predictor of QoL in this patient group as this represents a modifiable means by which individuals with renal impairment may improve their QoL and overall health perception.

These data reinforce the importance of routine screening for renal functional decline, such as in the monitoring of hypertension, since patients will not only be largely asymptomatic but are also unlikely to experience any subtle changes to health status.

## Conclusion

The present study has found no difference in QoL between individuals with existing CKD diagnoses, screen-detected CKD, and a transient GFR reduction. Age, smoking status and number of comorbid conditions predicted QoL in this CKD cohort, within which EQ-5D-5L detected more impairment in physical QoL domains than mental. Continued follow-up and larger prospective studies are required to define the relationship between QoL and CKD progression more precisely.

## Supporting information

S1 File Supporting information. (DOCX)

S1 Fig Flowchart of study design and participant subgroups. (TIF)

S1 Appendix OxRen case report form. (DOCX)
