# Supplementary material for: Quality of life in older adults with chronic kidney disease and transient changes in renal function: Findings from the Oxford Renal cohort
Source: PLoS One. 2022 Oct 14;17(10):e0275572. doi: 10.1371/journal.pone.0275572 (PMC9565742; doi:10.1371/journal.pone.0275572)
Supplement: S1 File — (DOCX) [file pone.0275572.s001.docx]

# **S1 File. Supporting Information**

|  | **Entire cohort (n=1063)** | **Existing CKD**  **(n=322)** | **Screen-detected CKD (n=480)** | **Transient eGFR reduction (n=261)** | **p-value^a^** |
| --- | --- | --- | --- | --- | --- |
| **Characteristics** | **Mean (SD)** | **Mean (SD)** | **Mean (SD)** | **Mean (SD)** |  |
| Age (years) | 74.51 (6.80) | 75.39 (6.98) | 74.33 (6.39) | 73.74 (7.21) | 0.011^*^ |
| Weight (kg) | 77.70 (16.70) | 77.64 (16.34) | 77.81 (16.92) | 77.57 (16.79) | 0.979 |
| Height (m) | 1.67 (0.10) | 1.67 (0.10) | 1.68 (0.10) | 1.66 (0.11) | 0.166 |
| Waist circ. (cm) | 96.61 (14.50) | 97.30 (13.51) | 96.19 (15.16) | 96.53 (14.47) | 0.568 |
| Hip circ. (cm) | 106.16 (10.46) | 107.00 (10.35) | 105.38 (10.46) | 106.53 (10.52) | 0.080 |
| BMI (kg/m^2^) | 27.78 (5.83) | 27.78 (5.14) | 27.58 (5.50) | 28.14 (7.09) | 0.467 |
| eGFR (ml/min/1.73m^2^) | 64.20 (15.44) | 56.38 (14.64) | 68.23 (14.50) | 67.07 (14.35) | <0.001^*^ |
| Number of comorbidities | 2.37 (1.48) | 3.03 (1.38) | 2.07 (1.48) | 2.09 (1.33) | <0.001^*^ |
|  | | | | | |
| **Sex** | **n (%)** | **n (%)** | **n (%)** | **n (%)** |  |
| Male | 488 (45.91) | 144 (44.72) | 237 (49.38) | 107 (41.00) | 0.088 |
| Female | 574 (54.00) | 178 (55.28) | 243 (50.63) | 153 (58.62) |  |
| **Ethnicity** | | | | | |
| White | 1048 (98.59) | 318 (98.76) | 475 (98.96) | 255 (97.70) | 0.598 |
| Other | 14 (1.41) | 4 (1.24) | 5 (1.04) | 5 (1.92) |  |
| **Comorbid disease** | | | | | |
| Hypertension | 615 (57.86) | 214 (66.46) | 257 (53.54) | 144 (55.17) | <0.001^*^ |
| Diabetes | 163 (15.33) | 72 (22.36) | 57 (11.88) | 34 (13.03) | <0.001^*^ |
| Ischaemic Heart Disease | 186 (17.50) | 63 (19.57) | 76 (15.83) | 47 (18.01) | 0.383 |
| Heart failure | 48 (4.52) | 19 (5.90) | 21 (4.38) | 8 (3.07) | 0.256 |
| Atrial Fibrillation | 130 (12.23) | 51 (15.84) | 47 (9.79) | 32 (12.26) | 0.038^*^ |
| Cerebrovascular disease | 87 (8.18) | 33 (10.25) | 38 (7.92) | 16 (6.13) | 0.189 |
| Peripheral vascular disease | 39 (3.67) | 9 (2.80) | 18 (3.75) | 12 (4.60) | 0.511 |
| Previous renal disease | 395 (37.16) | 263 (81.68) | 98 (20.42) | 34 (13.03) | <0.001^*^ |
| Anaemia | 115 (10.82) | 38 (11.80) | 43 (8.96) | 34 (13.03) | 0.186 |
| Osteoporosis | 86 (8.09) | 25 (7.76) | 45 (9.38) | 16 (6.13) | 0.292 |
| Osteopenia | 64 (6.02) | 22 (6.83) | 33 (6.88) | 9 (3.45) | 0.132 |
| **Smoking status** | | | | | |
| Never | 579 (54.47) | 166 (51.55) | 262 (54.58) | 151 (57.85) | 0.474 |
| Former | 438 (41.20) | 142 (44.10) | 194 (40.42) | 102 (39.08) |  |
| Current | 46 (4.33) | 14 (4.35) | 24 (5.00) | 8 (3.07) |  |
| **Educational status** | | | | | |
| No qualifications | 392 (36.88) | 124 (38.51) | 182 (37.92) | 86 (32.95) | 0.554 |
| GCSE/O-levels | 191 (17.97) | 57 (17.70) | 86 (17.92) | 48 (18.39) |  |
| A-levels | 208 (19.57) | 56 (17.39) | 90 (18.75) | 62 (23.75) |  |
| University/ Postgraduate | 272 (25.59) | 85 (26.40) | 122 (25.42) | 65 (24.90) |  |

**S1 Table. Baseline characteristics of the OxRen cohort of older adults from Oxfordshire primary care practices stratified by CKD subgroup.** a Variables compared between CKD subgroups; *p<0.05

**S2 Table. Baseline characteristics of the OxRen cohort of older adults from Oxfordshire primary care practices stratified by availability of complete QoL data.** ^a^ Variables compared between those with and without complete QoL data; ^*^p<0.05

|  | **Entire cohort (n=1063)** | **Complete QoL data (n= 857)** | **Incomplete/ missing QoL data (n= 206)** | **p-value^a^** |
| --- | --- | --- | --- | --- |
| **Characteristics** | **Mean (SD)** | **Mean (SD)** | **Mean (SD)** |  |
| Age (years) | 74.51 (6.80) | 74.52 (6.78) | 74.45 (6.93) | 0.893 |
| Weight (kg) | 77.70 (16.70) | 77.81 (16.74) | 77.25 (16.58) | 0.667 |
| Height (m) | 1.67 (0.10) | 1.67 (0.10) | 1.66 (0.11) | 0.236 |
| Waist circ. (cm) | 96.61 (14.50) | 96.80 (14.53) | 95.83 (14.36) | 0.391 |
| Hip circ. (cm) | 106.16 (10.46) | 106.23 (10.43) | 105.85 (10.60) | 0.634 |
| BMI (kg/m^2^) | 27.78 (5.83) | 27.70 (5.27) | 28.12 (7.75) | 0.345 |
| eGFR (ml/min/1.73m^2^) | 64.20 (15.44) | 63.79 (15.46) | 66.05 (15.22) | 0.092 |
| Number of comorbidities | 2.37 (1.48) | 2.37 (1.46) | 2.33 (1.54) | <0.001^*^ |
|  | | | | |
| **Sex** | **n (%)** | **n (%)** | **n (%)** |  |
| Male | 488 (45.91) | 400 (46.67) | 88 (42.72) | 0.338 |
| Female | 574 (54.00) | 456 (53.21) | 118 (57.28) |  |
| **Ethnicity** | | | | |
| White | 1048 (98.59) | 845 (98.60) | 203 (98.54) | 0.999 |
| Other | 14 (1.41) | 11 (1.28) | 3 (1.46) |  |
| **Comorbid disease** | | | | |
| Hypertension | 615 (57.86) | 503 (58.69) | 112 (54.37) | 0.294 |
| Diabetes | 163 (15.33) | 127 (14.82) | 36 (17.48) | 0.400 |
| Ischaemic Heart Disease | 186 (17.50) | 154 (17.97) | 32 (15.53) | 0.469 |
| Heart failure | 48 (4.52) | 37 (4.32) | 11 (5.34) | 0.654 |
| Atrial Fibrillation | 130 (12.23) | 107 (12.49) | 23 (11.17) | 0.689 |
| Cerebrovascular disease | 87 (8.18) | 72 (8.40) | 15 (7.28) | 0.700 |
| Peripheral vascular disease | 39 (3.67) | 31 (3.62) | 8 (3.88) | 0.999 |
| Previous renal disease | 395 (37.16) | 341 (39.79) | 54 (26.21) | <0.001* |
| Anaemia | 115 (10.82) | 99 (11.55) | 16 (7.77) | 0.148 |
| Osteoporosis | 86 (8.09) | 72 (8.40) | 14 (6.80) | 0.538 |
| Osteopenia | 64 (6.02) | 47 (5.48) | 17 (8.25) | 0.181 |
| **Smoking status** | | | | |
| Never | 579 (54.47) | 461 (53.79) | 118 (57.28) | 0.577 |
| Former | 438 (41.20) | 357 (41.66) | 81 (39.32) |  |
| Current | 46 (4.33) | 39 (4.55) | 7 (3.40) |  |
| **Educational status** | | | | |
| No qualifications | 392 (36.88) | 322 (37.57) | 70 (33.98) | 0.618 |
| GCSE/O-levels | 191 (17.97) | 148 (17.27) | 43 (20.87) |  |
| A-levels | 208 (19.57) | 168 (19.60) | 40 (19.42) |  |
| University/ Postgraduate | 272 (25.59) | 219 (25.55) | 53 (25.73) |  |

**S3 Table. QoL in the entire OxRen cohort of older adults from Oxfordshire primary care practices stratified by type of questionnaire, QoL dimension, and CKD subgroup.** ^1^ The sample size for the entire OxRen cohort or CKD subgroups may vary by questionnaire due to some participants having missing data for both or only EQ-5D-5L and ICECAP-A QoL questionnaires.
^2^ Ordered from level 1 to level 5 for EQ-5D-5L and from level 1 to level 4 for ICECAP-A.

|  | **EQ-5D-5L** | | | | |
| --- | --- | --- | --- | --- | --- |
| **Subgroup** ^1^  Domain Level ^2^ | **Mobility** | **Self-care** | **Usual activities** | **Pain/ Discomfort** | **Anxiety/ Depression** |
|  | **n (%)** | **n (%)** | **n (%)** | **n (%)** | **n (%)** |
| **Entire cohort (n=926)** | | | | | |
| No problem | 451 (49.7) | 825 (90.8) | 540 (59.3) | 278 (30.5) | 576 (63.5) |
| Slight problem | 279 (30.7) | 56 (6.2) | 255 (28.0) | 413 (45.3) | 256 (28.2) |
| Moderate problem | 144 (15.9) | 27 (3.0) | 101 (11.1) | 187 (20.5) | 64 (7.1) |
| Severe problem | 30 (3.3) | 1 (0.1) | 13 (1.4) | 31 (3.4) | 10 (1.1) |
| Unable to/ extreme | 4 (0.4) | 0 (0.0) | 2 (0.2) | 2 (0.2) | 1 (0.1) |
| **Existing CKD (n=298)** | | | | | |
| No problem | 130 (43.6) | 267 (89.6) | 171 (57.0) | 88 (29.2) | 194 (64.7) |
| Slight problem | 101 (33.9) | 18 (6.0) | 88 (29.3) | 138 (45.8) | 83 (27.7) |
| Moderate problem | 51 (17.1) | 12 (4.0) | 36 (12.0) | 60 (19.9) | 18 (6.0) |
| Severe problem | 13 (4.4) | 1 (0.3) | 5 (1.7) | 14 (4.7) | 4 (1.3) |
| Unable to/ extreme | 3 (1.0) | 0 (0.0) | 0 (0.0) | 1 (0.3) | 1 (0.3) |
| **Screen-detected CKD (n=383)** | | | | | |
| No problem | 204 (54.8) | 343 (92.2) | 233 (62.6) | 113 (30.5) | 236 (64.1) |
| Slight problem | 107 (28.8) | 18 (4.8) | 98 (26.3) | 182 (49.1) | 101 (27.4) |
| Moderate problem | 51 (13.7) | 11 (3.0) | 36 (9.7) | 64 (17.3) | 27 (7.3) |
| Severe problem | 9 (2.4) | 0 (0.0) | 3 (0.8) | 11 (3.0) | 4 (1.1) |
| Unable to/ extreme | 1 (0.3) | 0 (0.0) | 2 (0.5) | 1 (0.3) | 0 (0.0) |
| **Transient GFR reduction (n=243)** | | | | | |
| No problem | 117 (49.2) | 215 (90.0) | 136 (56.9) | 77 (32.2) | 146 (61.1) |
| Slight problem | 71 (29.8) | 20 (8.4) | 69 (28.9) | 93 (38.9) | 72 (30.1) |
| Moderate problem | 42 (17.6) | 4 (1.7) | 29 (12.1) | 63 (26.4) | 19 (7.9) |
| Severe problem | 8 (3.4) | 0 (0.0) | 5 (2.1) | 6 (2.5) | 2 (0.8) |
| Unable to/ extreme | 0 (0.0) | 0 (0.0) | 0 (0.0) | 0 (0.0) | 0 (0.0) |
|  | **ICECAP-A** | | | | |
|  | **Attachment** | **Stability** | **Achievement** | **Enjoyment** | **Autonomy** |
|  | **n (%)** | **n (%)** | **n (%)** | **n (%)** | **n (%)** |
| **Entire cohort (n= 888)** | | | | | |
| No capability | 8 (0.9) | 2 (0.2) | 0 (0.0) | 1 (0.1) | 2 (0.2) |
| Little capability | 47 (5.2) | 45 (5.0) | 28 (3.1) | 92 (10.3) | 54 (6.0) |
| Much capability | 449 (49.9) | 259 (28.9) | 287 (32.0) | 533 (59.6) | 391 (43.6) |
| Full capability | 396 (44.0) | 591 (65.9) | 583 (64.9) | 268 (30.0) | 450 (50.2) |
| **Existing CKD (n= 279)** | | | | | |
| No capability | 6 (2.1) | 1 (0.4) | 0 (0.0) | 0 (0.0) | 1 (0.4) |
| Little capability | 17 (6.0) | 10 (3.6) | 14 (5.0) | 31 (11.0) | 17 (6.0) |
| Much capability | 146 (51.8) | 78 (27.8) | 107 (37.9) | 179 (63.7) | 126 (44.8) |
| Full capability | 113 (40.1) | 192 (68.3) | 161 (57.1) | 71 (25.3) | 137 (48.8) |
| **Screen-detected CKD (n= 374)** | | | | | |
| No capability | 2 (0.5) | 0 (0.0) | 0 (0.0) | 1 (0.3) | 0 (0.0) |
| Little capability | 15 (4.0) | 16 (4.3) | 10 (2.6) | 38 (10.1) | 17 (4.5) |
| Much capability | 193 (51.1) | 111 (29.5) | 117 (31.0) | 220 (58.5) | 166 (44.0) |
| Full capability | 168 (44.4) | 249 (66.2) | 251 (66.4) | 117 (31.1) | 194 (51.5) |
| **Transient GFR reduction (n= 235)** | | | | | |
| No capability | 0 (0.0) | 1 (0.4) | 0 (0.0) | 0 (0.0) | 1 (0.4) |
| Little capability | 15 (6.3) | 19 (7.9) | 4 (1.7) | 23 (9.7) | 20 (8.4) |
| Much capability | 110 (45.8) | 70 (29.2) | 63 (26.5) | 134 (56.5) | 99 (41.4) |
| Full capability | 115 (47.9) | 150 (62.5) | 171 (71.8) | 80 (33.8) | 119 (49.8) |

**S4 Table. Secondary linear regression model, fully adjusted for all clinical and demographic variables. ^*^** p<0.05

|  | **N** | **Mean Utility Score** | **Coefficient** | **95% CI** | **p-value** |
| --- | --- | --- | --- | --- | --- |
|  | **EQ-5D-5L** | | | | |
| **CKD subgroup** | | | | | |
| Existing CKD | 480 | 0.763 | 0 Reference | | |
| Screen-detected CKD | 322 | 0.785 | 0.003 | -0.028 to 0.034 | 0.873 |
| Transient eGFR reduction | 261 | 0.779 | -0.001 | -0.035 to 0.032 | 0.937 |
| **Characteristics** | | | | | |
| Age (per 1 year) | 1063 | 0.776 | -0.004* | -0.006 to -0.002 | <0.001 |
| Sex (Female vs Male) | 574 | 0.768 | -0.046* | -0.084 to -0.009 | 0.015 |
| BMI | 1063 | 0.776 | -0.009 | -0.021 to 0.004 | 0.165 |
| White ethnicity | 1048 | 0.776 | 0.014 | -0.078 to 0.106 | 0.765 |
| eGFR (ml/min/1.73m^2^) | 1063 | 0.776 | 0.000 | -0.001 to 0.001 | 0.603 |
| **Comorbid disease** | | | | | |
| Diabetes | 163 | 0.728 | -0.032* | -0.063 to -0.001 | 0.046 |
| Hypertension | 615 | 0.768 | 0.002 | -0.021 to 0.024 | 0.893 |
| Ischaemic Heart Disease | 186 | 0.747 | -0.005 | -0.034 to 0.024 | 0.741 |
| Obesity | 321 | 0.733 | -0.028 | -0.066 to 0.009 | 0.142 |
| Hip circumference  (per 1 cm) | 1063 | 0.776 | 0.000 | -0.002 to 0.002 | 0.918 |
| Systolic Blood Pressure  (per 1 mmHg) | 1063 | 0.776 | 0.000 | 0.000 to 0.001 | 0.350 |
| Diastolic Blood Pressure  (per 1 mmHg) | 1063 | 0.776 | 0.000 | -0.001 to 0.002 | 0.701 |
| Alcohol (per 1 unit) | 1063 | 0.776 | 0.000 | -0.001 to 0.002 | 0.649 |
| Heart Failure | 48 | 0.713 | -0.037 | -0.091 to 0.017 | 0.174 |
| Atrial Fibrillation | 130 | 0.759 | 0.011 | -0.023 to 0.045 | 0.514 |
| Cerebrovascular disease | 87 | 0.703 | -0.068* | -0.106 to -0.030 | <0.001 |
| Peripheral vascular disease | 39 | 0.695 | -0.080* | -0.138 to -0.021 | 0.008 |
| Previous renal disease | 395 | 0.762 | -0.012 | -0.040 to 0.016 | 0.419 |
| Urinary Tract Infection | 455 | 0.748 | -0.048* | -0.071 to -0.025 | <0.001 |
| Thyroid | 132 | 0.763 | -0.007 | -0.040 to 0.025 | 0.665 |
| Anaemia | 115 | 0.734 | -0.026 | -0.060 to 0.008 | 0.137 |
| Osteopenia | 64 | 0.786 | 0.006 | -0.043 to 0.054 | 0.818 |
| Osteoporosis | 86 | 0.718 | -0.055* | -0.095 to -0.014 | 0.008 |
| **Smoking Status** | | | | | |
| Never smoked | 579 | 0.796 | 0 Reference | | |
| Former smoker | 438 | 0.755 | -0.023 | -0.046 to 0.000 | 0.047 |
| Current smoker | 46 | 0.721 | -0.097 | -0.150 to -0.043 | <0.001 |
| **Education Status** | | | | | |
| A-levels | 208 | 0.791 | 0 Reference | | |
| GCSEs/O-levels | 191 | 0.778 | 0.000 | -0.035 to 0.034 | 0.980 |
| University or Postgraduate | 272 | 0.818 | 0.016 | -0.017 to 0.049 | 0.338 |
| No qualifications | 392 | 0.739 | -0.029 | -0.059 to 0.001 | 0.060 |
|  | **ICECAP-A** | | | | |
| **CKD subgroup** | | | | | |
| Existing CKD | 480 | 0.895 | 0 Reference | | |
| Screen-detected CKD | 322 | 0.909 | 0.008 | -0.015 to 0.030 | 0.510 |
| Transient GFR reduction | 261 | 0.904 | 0.002 | -0.022 to 0.026 | 0.871 |
| **Characteristics** | | | | | |
| Age (per 1 year) | 1063 | 0.903 | -0.002 | -0.002 to -0.001 | 0.344 |
| Sex (Female vs Male) | 574 | 0.897 | -0.021 | -0.047 to 0.004 | 0.103 |
| BMI | 1063 | 0.903 | -0.005 | -0.010 to 0.001 | 0.102 |
| White ethnicity | 1048 | 0.904 | 0.073* | 0.015 to 0.132 | 0.014 |
| eGFR (ml/min/1.73m^2^) | 1063 | 0.903 | 0.000 | 0.000 to 0.001 | 0.141 |
| **Comorbid disease** | | | | | |
| Diabetes | 163 | 0.895 | -0.007 | -0.029 to 0.015 | 0.550 |
| Hypertension | 615 | 0.900 | 0.000 | -0.016 to 0.016 | 0.982 |
| Ischaemic Heart Disease | 186 | 0.885 | -0.020 | -0.040 to 0.001 | 0.056 |
| Obesity | 321 | 0.895 | 0.000 | -0.026 to 0.027 | 0.972 |
| Hip circumference  (per 1 cm) | 1063 | 0.903 | 0.000 | -0.001 to 0.002 | 0.641 |
| Systolic Blood Pressure  (per 1 mmHg) | 1063 | 0.903 | 0.000 | -0.001 to 0.000 | 0.533 |
| Diastolic Blood Pressure  (per 1 mmHg) | 1063 | 0.903 | 0.000 | -0.001 to 0.001 | 0.516 |
| Alcohol (per 1 unit) | 1063 | 0.903 | 0.001 | 0.000 to 0.002 | 0.124 |
| Heart Failure | 48 | 0.897 | 0.012 | -0.025 to 0.049 | 0.531 |
| Atrial Fibrillation | 130 | 0.905 | 0.015 | -0.009 to 0.039 | 0.212 |
| Cerebrovascular disease | 87 | 0.871 | -0.041^*^ | -0.067 to -0.014 | 0.003 |
| Peripheral vascular disease | 39 | 0.868 | -0.045^*^ | -0.086 to -0.003 | 0.035 |
| Previous renal disease | 395 | 0.899 | 0.003 | -0.017 to 0.023 | 0.758 |
| Urinary Tract Infection | 455 | 0.891 | -0.019^*^ | -0.035 to -0.03 | 0.021 |
| Thyroid | 132 | 0.903 | -0.001 | -0.025 to 0.022 | 0.906 |
| Anaemia | 115 | 0.893 | 0.003 | -0.021 to 0.027 | 0.806 |
| Osteopenia | 64 | 0.886 | -0.024 | -0.058 to 0.011 | 0.179 |
| Osteoporosis | 86 | 0.880 | -0.016 | -0.045 to 0.012 | 0.260 |
| **Smoking status** | | | | | |
| Never smoked | 579 | 0.9114 | 0 Reference | | |
| Former smoker | 438 | 0.8961 | -0.012 | -0.028 to 0.004 | 0.151 |
| Current smoker | 46 | 0.8752 | -0.042^*^ | -0.079 to -0.005 | 0.027 |
| **Education status** | | | | | |
| A-levels | 208 | 0.919 | 0 Reference | | |
| GCSEs/O-levels | 191 | 0.919 | 0.003 | -0.021 to 0.027 | 0.803 |
| University or Postgraduate | 272 | 0.915 | -0.011 | -0.034 to 0.012 | 0.357 |
| No qualifications | 392 | 0.880 | -0.033* | -0.054 to -0.012 | 0.002 |

**S5 Table. Adjusted estimates of the association of CKD stage with QoL utility score.** ^*^ p<0.05

| **CKD stage** | **eGFR threshold (ml/min/1.73m^2^)** | **N** | **Coefficient** | **95% CI** | **p-value** |
| --- | --- | --- | --- | --- | --- |
| **EQ-5D-5L** | | | | | |
| 1 | ≥90 | 43 | 0 Reference | | |
| 2 | 60-89 | 506 | -0.042 | -0.100, 0.014 | 0.139 |
| 3a | 45-59 | 277 | -0.076 | -0.133, -0.019 | 0.009^*^ |
| 3b | 30-44 | 95 | -0.107 | -0.171, -0.042 | 0.001^*^ |
| 4 | 15-29 | 8 | -0.064 | -0.196, 0.067 | 0.336 |
| **ICECAP-A** | | | | | |
| 1 | ≥90 | 43 | 0 Reference | | |
| 2 | 60-89 | 506 | 0.003 | -0.033, 0.039 | 0.863 |
| 3a | 45-59 | 277 | -0.012 | -0.049, 0.026 | 0.541 |
| 3b | 30-44 | 95 | -0.031 | -0.073, 0.011 | 0.144 |
| 4 | 15-29 | 8 | 0.017 | -0.069, 0.103 | 0.700 |

**S6 Table. Sensitivity analyses for QoL questionnaire data.** **Vif>5.000 and GVIF^1/(2-df)^>2.236 indicate collinearity.

|  | **Variance Inflation Factor** | **Degrees of Freedom (df)** | **GVIF^1/(2-df)^** |
| --- | --- | --- | --- |
| **EQ-5D-5L** | | | |
| CKD subgroup | 1.852 | 2 | 1.167 |
| Age (per 1 year) | 1.538 | 1 | 1.240 |
| Sex | 3.213 | 1 | 1.793 |
| BMI (per 1 kg/m^2^) | 39.198^**^ | 1 | 6.261^**^ |
| Diabetes | 1.168 | 1 | 1.081 |
| Hypertension | 1.152 | 1 | 1.073 |
| Ischaemic Heart Disease | 1.156 | 1 | 1.075 |
| Smoking Status | 1.198 | 2 | 1.046 |
| Weight (per 1 kg) | 61.267^**^ | 1 | 7.827^**^ |
| Height (per 1 cm) | 22.115^**^ | 1 | 4.703^**^ |
| Obesity | 2.821 | 1 | 1.680 |
| Waist circumference (per 1 cm) | 6.359^**^ | 1 | 2.522^**^ |
| Hip circumference  (per 1 cm) | 4.223 | 1 | 2.055 |
| Systolic Blood Pressure  (per 1 mmHg) | 1.946 | 1 | 1.395 |
| Diastolic Blood Pressure  (per 1 mmHg) | 1.920 | 1 | 1.386 |
| Alcohol (per 1 unit) | 1.169 | 1 | 1.081 |
| Heart Failure | 1.112 | 1 | 1.054 |
| Atrial Fibrillation | 1.174 | 1 | 1.083 |
| Cerebrovascular disease | 1.108 | 1 | 1.053 |
| Peripheral vascular disease | 1.067 | 1 | 1.033 |
| Previous renal disease | 1.731 | 1 | 1.316 |
| Urinary Tract Infection | 1.189 | 1 | 1.090 |
| Thyroid | 1.076 | 1 | 1.037 |
| Anaemia | 1.071 | 1 | 1.035 |
| Osteopenia | 1.103 | 1 | 1.050 |
| Osteoporosis | 1.087 | 1 | 1.043 |
| Ethnicity | 1.058 | 1 | 1.029 |
| Education Status | 1.302 | 3 | 1.045 |
| eGFR (ml/min/1.73m^2^) | 1.373 | 1 | 1.172 |
| **ICECAP-A** | | | |
| CKD subgroup | 1.982 | 2 | 1.187 |
| Age (per 1 year) | 1.519 | 1 | 1.232 |
| Sex | 3.119 | 1 | 1.766 |
| BMI (per 1 kg/m^2^) | 20.017^**^ | 1 | 4.474^**^ |
| Diabetes | 1.176 | 1 | 1.084 |
| Hypertension | 1.162 | 1 | 1.078 |
| Ischaemic Heart Disease | 1.147 | 1 | 1.071 |
| Smoking Status | 1.214 | 2 | 1.050 |
| Weight (per 1 kg) | 38.368^**^ | 1 | 6.194^**^ |
| Height (per 1 cm) | 14.413^**^ | 1 | 3.796^**^ |
| Obesity | 2.821 | 1 | 1.680 |
| Waist circumference (per 1 cm) | 6.079^**^ | 1 | 2.465^**^ |
| Hip circumference  (per 1 cm) | 3.770 | 1 | 1.942 |
| Systolic Blood Pressure  (per 1 mmHg) | 1.968 | 1 | 1.403 |
| Diastolic Blood Pressure  (per 1 mmHg) | 1.968 | 1 | 1.403 |
| Alcohol (per 1 unit) | 1.185 | 1 | 1.089 |
| Heart Failure | 1.100 | 1 | 1.049 |
| Atrial Fibrillation | 1.161 | 1 | 1.078 |
| Cerebrovascular disease | 1.114 | 1 | 1.055 |
| Peripheral vascular disease | 1.077 | 1 | 1.038 |
| Previous renal disease | 1.822 | 1 | 1.350 |
| Urinary Tract Infection | 1.177 | 1 | 1.085 |
| Thyroid | 1.079 | 1 | 1.039 |
| Anaemia | 1.079 | 1 | 1.039 |
| Osteopenia | 1.081 | 1 | 1.040 |
| Osteoporosis | 1.091 | 1 | 1.044 |
| Ethnicity | 1.054 | 1 | 1.027 |
| Education Status | 1.321 | 3 | 1.048 |
| eGFR (ml/min/1.73m^2^) | 1.405 | 1 | 1.185 |
