# Supplementary figures and images for: Quality of life in older adults with chronic kidney disease and transient changes in renal function: Findings from the Oxford Renal cohort
Source: PLoS One. 2022 Oct 14;17(10):e0275572. doi: 10.1371/journal.pone.0275572 (PMC9565742; doi:10.1371/journal.pone.0275572)

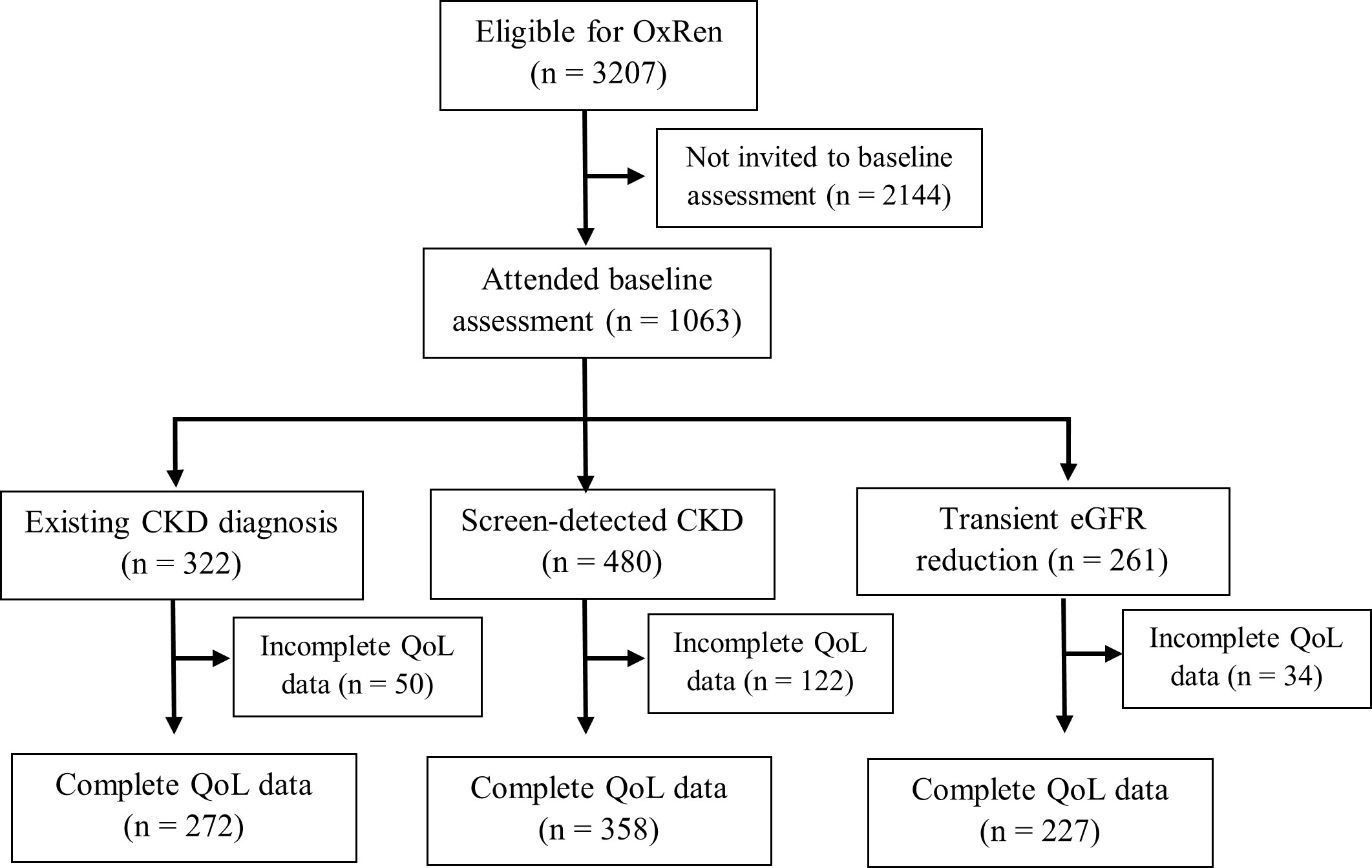

Supplement: S1 Fig — (TIF) [file pone.0275572.s002.tif]
