# Supplementary material for: Quality of life in older adults with chronic kidney disease and transient changes in renal function: Findings from the Oxford Renal cohort
Source: PLoS One. 2022 Oct 14;17(10):e0275572. doi: 10.1371/journal.pone.0275572 (PMC9565742; doi:10.1371/journal.pone.0275572)
Supplement: S1 Appendix — (DOCX) [file pone.0275572.s003.docx]

**S1 Appendix. OxRen case report form**

| DESCRIPTION_LABEL | RESPONSE_OPTIONS_TEXT | | | RESPONSE_VALUES | |
| --- | --- | --- | --- | --- | --- |
| Visit Date |  | | |  | |
| Date of full informed consent |  | | |  | |
| Participant ID (Enter in uppercase only) |  | | |  | |
| Date of Birth |  | | |  | |
| **1. DEMOGRAPHICS** |  | | |  | |
| Ethnicity | 1. White - English / Welsh / Scottish / Northern Irish / British,2. White - Irish,3. White - Gypsy or Irish Traveller,4. White - Any other White background\\, please describe,5. Mixed / Multiple ethnic groups - White and Black Caribbean,6. Mixed / Multiple ethnic groups - White and Black African,7. Mixed / Multiple ethnic groups - White and Asian,8. Mixed / Multiple ethnic groups - Any other Mixed / Multiple ethnic background\\, please describe,9. Asian / Asian British - Indian,10. Asian / Asian British - Pakistani,11. Asian / Asian British - Bangladeshi,12. Asian / Asian British - Chinese,13. Asian / Asian British - Any other Asian background\\, please describe,14. Black / African / Caribbean / Black British - African,15. Black / African / Caribbean / Black British - Caribbean,16. Black / African / Caribbean / Black British - Any other Black / African / Caribbean background\\, please describe,17. Other ethnic group - Arab,18. Other ethnic group - Any other ethnic group\\, please describe | | | 1,2,3,4,5,6,7,8, 9,10,11,12,13, 14,15,16,17,18 | |
| Ethnicity - Other |  | | |  | |
| Gender | Female,Male | | | 0,1 | |
| Postcode |  | | |  | |
| **2. PHYSICAL EXAMINATION/ VITAL SIGNS** | | |  | |  |
| Weight |  | |  | |  |
| Height |  | | |  | |
| Waist |  | | |  | |
| Hip |  | | |  | |
| Blood Pressure 1 Systolic |  | | |  | |
| Blood Pressure 1 Diastolic |  | | |  | |
| Blood Pressure 2 Systolic |  | | |  | |
| Blood Pressure 2 Diastolic |  | | |  | |
| Blood Pressure 3 Systolic |  | | |  | |
| Blood Pressure 3 Diastolic |  | | |  | |
| ECG performed? | Yes,No | | | 1,0 | |
| Please provide ECG date |  | | |  | |
| PWA Measurement |  | | |  | |
| **3. SUBSTANCE USE** |  |  |  |  |  |
| What is the participants smoking status? | Never,Current,Former | | | 0,1,2 | |
| What is the participants alcohol intake? |  | | |  | |
| **4. PARTICIPANT CHARACTERISTICS** |  |  |  |  |  |
| What is the participants highest education level achieved? | School to 16 no qualifications,School to 16\\, GCSEs/O levels,Sixth Form school or college\\, A Levels\\, ND,Highers\\, Scotvec or NVQ,University degree,Professional or postgraduate degree | | | 0,1,2,3,4,5 | |
| **5. MEDICAL HISTORY** |  |  |  |  |  |
| Hypertension - start date |  | | |  | |
| Diabetes - start date |  | | |  | |
| Diabetes impaired fasting glucose AND/OR impaired glucose tolerance |  | | |  | |
| Ischaemic heart disease | Myocardial infarct,Angina onset,Angioplasty +/- stent,Coronary artery bypass graft,Other | | | 1,2,3,4,5 | |
| IHD - start date |  | | |  | |
|  |  | | |  | |
| Heart failure - type | Reduced ejection fraction,Preserved ejection fraction,Other | | | 1,2,3 | |
| Heart failure - start date |  | | |  | |
|  |  | | |  | |
| Atrial fibrillation - type | Paroxysmal,Persistent,Other | | | 1,2,3 | |
| Atrial fibrillation - start date |  | | |  | |
| Cerebrovascular disease | Transient ischaemic attack,Ischaemic stroke,Haemorrhagic stroke,Other | | | 1,2,3,4 | |
| Cerebrovascular disease - start date |  | | |  | |
| Peripheral vascular disease - type | Claudication,Acute ischaemic limb,Angioplasty +/- stent,Endarterectomy,Other | | | 1,2,3,4,5 | |
| Peripheral vascular disease - start date |  | | |  | |
| Previous renal disease - type | Renal stones,Renal cysts,Nephrotic syndrome,Glomerulonephritis,Other | | | 1,2,3,4,5 | |
| Previous renal disease - start date |  | | |  | |
| Urinary Tract Infection - Childhood (UTI) | Yes,No,Unknown | | | 1,0,9 | |
| Urinary Tract Infection - Adulthood (UTI) |  | | |  | |
| Thyroid disease - start date |  | | |  | |
| Thyroid disease - type |  | | |  | |
| Anaemia - start date |  | | |  | |
| Anaemia - cause |  | | |  | |
| Osteopenia - start date |  | | |  | |
| Osteoporosis - start date |  | | |  | |
| **7. LABORATORY TEST COLLECTION** | | | |  | |
| Blood Sample collected? | YN | | |  | |
| If yes, please provide collection date |  | | |  | |
| Urine (ACR) Sample collected? | YN | | |  | |
| If yes, please provide collection date |  | | |  | |
| Time of Urine Collection | First Morning (before eating),Morning (before 12 noon),Afternoon (12-4 pm),Evening (after 4pm) | | | 1,2,3,4 | |
| Accession/Serial Number |  | | |  | |
| **8. PARTICIPANT COMPLETED QUESTIONNAIRE COLLECTION** | | | |  | |
| Quality of Life Questionnaire | YN | | |  | |
| Diet Questionnaire | YN | | |  | |
| Physical Activity Questionnaire | YN | | |  | |
| Cognitive Assessment Questionnaire | YN | | |  | |
